# Validation, Optimization, and Application of the Zebrafish Developmental Toxicity Assay for Pharmaceuticals Under the ICH S5(R3) Guideline

**DOI:** 10.3389/fcell.2021.721130

**Published:** 2021-09-14

**Authors:** Yi-Sheng Song, Ming-Zhu Dai, Chen-Xia Zhu, Yan-Feng Huang, Jing Liu, Cheng-Da Zhang, Feng Xie, Yi Peng, Yong Zhang, Chun-Qi Li, Li-Jiang Zhang

**Affiliations:** ^1^Center of Safety Evaluation and Research, Hangzhou Medical College, Hangzhou, China; ^2^Key Laboratory of Drug Safety Evaluation and Research of Zhejiang Province, Hangzhou Medical College (Zhejiang Academy of Medical Sciences), Hangzhou, China; ^3^Hunter Biotechnology, Inc., Hangzhou, China; ^4^Collaborative Innovation Center of Yangtze River Delta Region Green Pharmaceuticals, Zhejiang University of Technology, Hangzhou, China

**Keywords:** zebrafish, developmental toxicity, teratogenicity, malformation, embryo-fetal lethality (MEFL), alternative method, ICH S5(R3)

## Abstract

The zebrafish as an alternative animal model for developmental toxicity testing has been extensively investigated, but its assay protocol was not harmonized yet. This study has validated and optimized the zebrafish developmental toxicity assay previously reported by multiple inter-laboratory studies in the United States and Europe. In this study, using this classical protocol, of 31 ICH-positive compounds, 23 compounds (74.2%) were teratogenic in zebrafish, five had false-negative results, and three were neither teratogenic nor non-teratogenic according to the protocol standard; of 14 ICH-negative compounds, 12 compounds (85.7%) were non-teratogenic in zebrafish and two had false-positive results. After we added an additional TI value in the zebrafish treated with testing compounds at 2 dpf along with the original 5 dpf, proposed a new category as the uncategorized compounds for those TI values smaller than the cutoff both at 2 dpf and 5 dpf but inducing toxic phenotypes, refined the testing concentration ranges, and optimized the TI cut-off value from ≥ 10 to ≥ 3 for compounds with refined testing concentrations, this optimized zebrafish developmental assay reached 90.3% sensitivity (28/31 positive compounds were teratogenic in zebrafish) and 88.9% (40/45) overall predictability. Our results from this study strongly support the use of zebrafish as an alternative *in vivo* method for screening and assessing the teratogenicity of candidate drugs for regulatory acceptance.

## Introduction

Developmental toxicity and teratogenicity represent a severe safety problem that causes approximately 5–10% of the congenital abnormalities of human newborns by teratogenic agents (Seiler et al., 2009). After the developmental effects of thalidomide were recognized in 1966, the Food and Drug Administration (FDA) established protocols to be used for assessing drug effects on reproduction and development prior to approval for human use (Marathe and Thomas, 1990). In addition, there are currently more than 143,835 preregistered chemicals that could contaminate food and the environment (Rovida and Hartung, 2009; Kim et al., 2016), but about 86% of these and other existing chemicals have no safety testing data (Selderslaghs et al., 2012).

Due to concerns about the safety of chemicals, the European Registration, Evaluation, Authorization and restriction of Chemicals (REACH) program established protocols to collect such data for all chemicals produced or marketed in quantities of more than 1 t per year (Selderslaghs et al., 2012). It was estimated that 5816 traditional testing animals and a £1,883,200 cost would be required for the assessment of the developmental toxicity of one chemical according to the Organization for Economic Co-operation and Development (OECD) guidelines TG 414 (Organization for Economic Co-operation, and Development [OECD], 2001a), TG 416 (Organization for Economic Co-operation, and Development [OECD], 2001b), TG 421 (Organization for Economic Co-operation and Development [OECD], 1995), and TG 422 (Organization for Economic Co-operation and Development [OECD], 1996), no matter how time-consuming (Fleischer, 2007). This situation has urged research into alternative methods for developmental toxicity testing, namely, the whole embryo culture (WEC) test (Webster et al., 1997), the mammalian micromass (MM) test (Flint, 1993), and the embryonic stem cell test (EST; Spielmann et al., 1997). The former two models still use intact mammals to serve as test systems; the last one has always been a controversial area. Indeed, these tests do not cover the whole period of embryo development (Spielmann et al., 2006).

The zebrafish as a non-mammalian vertebrate animal furnishes several advantages for alternative toxicity assay, such as economic husbandry requirements, embryonal transparency, high fecundity, and 6-384-well plate high-throughput screening (Brannen et al., 2010; Lantz-Mcpeak et al., 2015). Furthermore, zebrafish also appears to be an applicable model for developmental toxicity testing, and the developmental toxicity of drugs and compounds has actually been evaluated in zebrafish in the past 10 years as summarized in Table 1 (Organization for Economic Co-operation, and Development [OECD], 2011; Organization for Economic Co-operation, and Development [OECD], 2012). In one blinded study in four laboratories, 20 non-proprietary compounds were tested in zebrafish for developmental toxicity; each of the testing laboratories achieved similar overall concordance to the mammalian data (60–70%; Gustafson et al., 2012). After optimizing experimental parameters and taking zebrafish embryo-uptake into consideration, in their second phase of this project, 38 proprietary pharmaceutical compounds were evaluated in two laboratories; 62–82% total concordance was achieved (Ball et al., 2014). In other studies, the zebrafish developmental toxicity assay achieved an overall predictive value of 50–60% and 92% at Flemish Institute for Technological Research and at Phylonix Pharmaceutical, respectively (Haldi et al., 2011; Selderslaghs et al., 2012). It was easy to find that these developmental toxicity reports derived from the zebrafish assays were not uniform: the definition of teratogenicity, predictability of results, multiple details related to experimental conditions and data analysis, and rationality of concentration settings were not fully optimized and validated.

**TABLE 1:** Published developmental toxicity of compounds evaluated in zebrafish.

**Time**	**Laboratory**	**Zebrafish lines**	**Exposure period**	**Evaluation index**	**Predictability/test compounds**	**Notes**
2011	OECD	–	Newly fertilized eggs were exposed for 96 h	Coefficients of variation, LC_50_	–/7	Experiments were evaluated in 7 laboratories
2012	OECD	–	Newly fertilized eggs were exposed for 96 h	Coefficients of variation, LC_50_	–/13	Experiments were evaluated in 4 laboratories
2012	AstraZeneca et al.	Wild type and WIK strain	6 hpf-5 dpf	Morphological scoring, LC_25_, LOAEL, NOAEL	60–70%/20	Experiments were evaluated blinded in 4 laboratories
2012	FITR^*^	Wild type	2 hpf-6 dpf	TI(= LC_50_//EC_50_)	50–60%/27	Experiments were conducted for 3 times
2012	Phylonix, Cambridge et al.	–	6 hpf-5 dpf	TI(= LC_50_//EC_50_)	92%/12	–
2014	AstraZeneca et al.	Wild type	6 hpf-5 dpf	Morphological scoring, LOAEL, TI(= LC_25_/NOAEL)	62–82%/38	Experiments were evaluated blinded in 2 laboratories
2014	FDA	Hb9: GFP transgenic zebrafish	24 hpf-52 hpf	Body length, fiber length	–/5	–

^*^Flemish Institute for Technological Research.

Publication and implementation of the International Conference of Harmonization (ICH) Harmonized Guideline S5(R3) (International Conference on Harmonization [ICH], 2017, 2020) is driving the progress of alternative methods for developmental toxicity testing in China. The introduction of alternative test systems in the International Conference on Harmonization (ICH) S5(R3) step 2 draft guideline (2017) (International Conference on Harmonization [ICH], 2017) of which China participated in the revision is one of the biggest updates, and it is also the first time that the content of alternative test methods has been added at great length in the ICH safety evaluation guidelines. The ICH guidelines proposed the use of alternative *in vitro* and non-mammalian *in vivo* reproduction tests for embryo-fetal developmental toxicity (EFD) risk assessment. The ICH S5(R3) final version (2020) has clearly stated that data generated from qualified alternative assays conducted alone or in conjunction with one or more *in vivo* studies can be utilized to support hazard identification and risk assessment under limited circumstances. However, the data produced from the zebrafish developmental toxicity assay have been used for investigative new drug (IND) applications domestically and internationally but not officially recognized by China drug regulatory agency yet.

In this study, we intended to validate and optimize internationally the preliminarily established the zebrafish developmental toxicity assay as an alternative *in vivo* assay, and hopefully for the regulatory acceptance of a China drug regulatory agency. Following the recommendation of the ICH Reference Compound List (Accessory 11.3.4, Table 9-6 Reference Compounds for Qualifying Alternative Assays, S5(R3) step 2 draft guideline, International Conference on Harmonization [ICH], 2017), 45 compounds were selected from among the 66 compounds based on their categories for the teratogenicity experiments: 31 out of 50 positive controls and 14 out of 16 negative controls. We have assessed and validated the zebrafish developmental toxicity assay protocol originally reported by multiple interlaboratory studies (Gustafson et al., 2012; Ball et al., 2014), here designated as the “classic protocol.” Combining our experience (He et al., 2014) and that of other studies (Haldi et al., 2011; Selderslaghs et al., 2012), we have optimized the original classical protocol and further improved its predictability for pharmaceutical use under the ICH S5(R3) guideline.

Additionally, three non-ICH reference compounds, chlorogenic acid, triptolide, and aconitine, were applied to test the optimum zebrafish developmental toxicity assay. The results derived from this study indicated that the zebrafish developmental toxicity assay optimized and validated in this report is a reliable and reproducible non-mammalian *in vivo* method for screening and assessing the teratogenicity (i.e., Malformations or Embryo-Fetal Lethality, MEFL) of candidate compounds.

## Materials and Methods

### Zebrafish Husbandry and Egg Collection

Adult AB strain zebrafish were obtained from the China Zebrafish Resource Center (Shanghai, China) and housed in a light- and temperature-controlled aquaculture facility with a standard 14:10-h light/dark photoperiod and fed with live brine shrimp twice daily and dry flake once a day. Four to five pairs of zebrafish were set up for natural mating every time. On average, 200–300 embryos were generated. Embryos were maintained at 28°C in fish water (0.2% Instant Ocean Salt in deionized water, pH 6.9–7.2, conductivity 480–510 mS.cm^–1^ and hardness 53.7–71.6 mg/L CaCO_3_). The embryos were washed and staged at 6 and 24 h post-fertilization (hpf) (Kimmel et al., 1995). The zebrafish facility at Hunter Biotechnology, Inc., is accredited by the Association for Assessment and Accreditation of Laboratory Animal Care (AAALAC) International (No. 001458,Zhou et al., 2015; Zhu et al., 2016), the China National Accreditation Service for Conformity Assessment (CNAS), and the China Inspection Body and Laboratory Mandatory Approval (CMA). After experiments, all the zebrafish were anesthetized and euthanized with 0.25 g/L tricaine methanesulfonate, which conforms to the American Veterinary Medical Association (AVMA) requirements for euthanasia by anesthetic (Shen et al., 2015). This study was approved by the Institutional Animal Care and Use Committee (IACUC) at Hunter Biotechnology, Inc., and the IACUC approval numbers were IACUC-2018-017, IACUC-2018-043, and IACUC-2020-117.

### Selection of Test Compounds

The 45 designated-compounds, in the ICH Reference Compound List, were selected following the ICH S5(R3) Step 2 Draft (2017) that stated, “to be appropriate for regulatory use, the alternative assay(s) should be characterized using the ICH Reference Compound List”; “at least 45 compounds in total should be tested”; and “all classes should be tested (at least two or three compounds from each class). An approximate 2:1 ratio of positive to negative compounds should be tested because it is important to identify positive compounds.” These ICH-positive (teratogenic) and -negative (non-teratogenic) compounds were evaluated for the validation of the proposed zebrafish developmental toxicity assay (Gustafson et al., 2012; Ball et al., 2014) in this study. These ICH reference compounds were classified into 10 categories based on their action mechanisms and summarized in Supplementary Table 1. All these compounds were ICH-approved drugs used clinically for patients, and developmental toxicity and teratogenicity have been confirmed in humans or mammalian animals [step 2 draft ICH harmonized guidelines S5(R3), 2017; Gustafson et al., 2012]. These tested reference compounds were purchased from Shanghai Aladdin Bio-Chem Technology Co., Ltd. (Aladdin) or Wuhan Dahua Pharmaceutical Co., Ltd. (WD Pharma), China (Supplementary Table 1; chemical properties of these compounds are shown in Supplementary Table 6). Dimethylsulfoxide (DMSO; Lot # SHBH6853, purity ≥ 99.8%) was purchased from Sigma.

The compounds triptolide (lot # Y29S9H71524; Shanghai Yuanye Biological Technology Co., Ltd., China) and aconitine (lot # 00001340-718; Chromadex, United States) are marketed drugs, and chlorogenic acid (lot # ZZS19041904; Shanghai Zhenzhu Biotechnology Co., Ltd., China) is a new drug candidate under pre-clinical development in China. These three compounds were not in the ICH Reference Compound List, and their developmental toxicity and teratogenicity in zebrafish were unknown.

The compounds were dissolved in 100% DMSO, and subsequently, various volumes of the master solutions were directly added to the testing fish water at designated testing concentrations with a final DMSO concentration of 0.5% (v/v). All the master solutions were prepared freshly right before each experiment, and pH values were checked without any artificial adjustments.

### Compound Treatment

The evaluation of malformation and mortality in zebrafish after exposure to compounds was performed following the previous reports from multiple inter-laboratory studies (Gustafson et al., 2012; Ball et al., 2014; Raghunath et al., 2019). At 4–6 hpf, zebrafish were manually transferred into a 24-well plate (Nest Biotech, Shanghai, China) containing negative compounds, positive compounds, or vehicle (0.5% DMSO), with 12 zebrafish per well in 1 ml of fish water. We selected zebrafish treatment starting at 4–6 h because most unfertilized zebrafish eggs could be easily identified and removed at 4 hpf and most zebrafish laboratories in China and Euro-America perform zebrafish egg cleaning and staging at 4–6 hpf. As reported in our previous studies and others (He et al., 2014), using 4–6 hpf high-quality fertilized zebrafish eggs for developmental toxicity assay would produce more reliable results. Untreated (fish water) control zebrafish were examined in parallel. Uniformly, a range of five concentrations at 0.1, 1, 10, 100, and 1000 μM were tested to assess the teratogenicity of a test compound. Exposure was continuous and static without feeding; dead zebrafish were recorded and removed from the solution during daily observations, and morphological characteristics of each individual zebrafish were evaluated at 48 hpf (2 days post-fertilization, dpf) and at 120 hpf (5 dpf) under a stereomicroscope (Nikon, SMZ645, Tokyo, Japan). False-positive, false-negative, and uncategorized compounds were repeated for at least one time.

### Determination of LC_25_ and NOAEL

Accumulated mortality was counted at 2 dpf and 5 dpf to calculate 25% lethal concentration (LC_25_), respectively, based on lethality curve. If lethality at the highest tested concentration was <25%, an LC_25_ value was by default set to be higher than the highest tested concentration in subsequent applications (Gustafson et al., 2012). Determination of the no observed adverse effect level (NOAEL) involved assessing the concentration–response relationship of abnormal effects; an adverse effect must be a gradient concentration-response. The NOAEL exhibited compound-related anomalies equal to or below those observed in the vehicle and negative controls. There were two NOAELs obtained from 2 dpf and 5 dpf observations, respectively, under a stereomicroscope.

### Developmental Toxicity Assessment

In the original classical protocol, zebrafish developmental toxicity was only assessed at 5 dpf, which led to higher false-negative results. In our pilot study, we found that zebrafish developmental toxicity assessed at both 2 dpf and 5 dpf could give a better predictability for positive teratogenic compounds. After compound treatment, zebrafish were immobilized using 3% methylcellulose and photographed for morphological anomalies using a 1 × DF PLAPO objective (Olympus, Japan) and a VertA1 camera (Sony Exmor CCD Sensor). Developmental malformations of structure/organ in morphology observed in zebrafish are summarized in Table 2. The assessed tissues and organs included heart, circulation, hemorrhage/thrombosis, head, pharyngeal arches/jaw, eye, sacculi/otoliths, liver, kidney, swim bladder, intestinal tract, notochord/somites/tail, trunk muscle, pectoral fins, and body pigmentation (Haldi et al., 2011; Gustafson et al., 2012; Ball et al., 2014; He et al., 2014). In the previous work, we found that swim bladder loss or delayed yolk sac absorption happened quite a lot in zebrafish embryos exposed to various pharmaceuticals and environmental agents, not specific for developmental toxicity. Therefore, swim bladder loss and yolk sac absorption were recorded in Table 2, but not used for developmental toxicity assessment and TI calculation.

**TABLE 2:** Developmental malformations observed in zebrafish.

**Structure/organ**	**Criteria of common developmental malformations**
Heart	Pericardial edema, bradycardia, arrhythmia (atrioventricular ratio) or missing chambers
Circulation	Absent, slow, or fast blood flow, defects in circulatory pattern
Hemorrhage/thrombosis	Presence of pooled blood outside of vascular network, a stagnant blood flow/a blood clot in the cardinal vein
Head	Malformation, abnormal size, degeneration
Pharyngeal arches/jaw	Oversized
Eye	Malformation, abnormal size
Sacculi/otoliths	Absent or small
Liver	Absent or abnormal size, degeneration (dark brown, opaque tissue), delayed yolk sac absorption
Kidney	Renal edema
Swim bladder	Missing
Intestinal tract	Absent, no or oversized lumen, does not extend to anal pore
Notochord/somites/tail	Shriveled tail fins
Trunk muscle	Muscle texture disordered or irregular
Pectoral fins	Absent or small
Body pigmentation	Significantly higher or lower amount of black pigment

### Teratogenicity and Teratogenic Index Calculation

#### STEP 1: Validation of the Classical Protocol (Gustafson et al., 2012; Ball et al., 2014)

Based on NOAEL and LC_25_ values, a teratogenic index (TI) was calculated as the ratio of LC_25_/NOAEL for each time point (Gustafson et al., 2012; Ball et al., 2014). In this classical protocol, compounds were classified as non-teratogens or teratogens according to the TI values derived from 5 dpf. A TI value in 5 dpf greater than or equal to 10 represented abnormalities for which compounds were predicted as teratogens; if TI values were <10, the compounds were predicted as non-teratogens; and compounds for which the LC_25_-to-NOAEL ratio could not be determined due to both concentrations being >1000 μM were predicted as non-teratogens (Gustafson et al., 2012; Ball et al., 2014).

#### STEP 2: Optimizations of the Test Concentration Range and Teratogenic Criteria

The optimizations of the classical protocol included calculating an additional TI value at 2 dpf, adding a new category of the uncategorized compounds, narrowing down the testing concentration ranges for some compounds identified in the initial tests and proposing a new teratogenic criterion.

(1) The calculating method of the classical protocol based on LC_25_ and NOAEL was used to obtain a TI value for compound-treated zebrafish at 2 dpf, and a TI value from either 2 dpf or 5 dpf greater than or equal to 10 represented a teratogenic compound. To reduce possible false-negative results, compounds that showed obvious morphological effects on zebrafish, but with TI values <10, were categorized as uncategorized teratogens, in which toxic potential could cover up the teratogenic potency.
(2) Uncategorized and false-positive compounds identified in the initial tests were further re-tested using the smaller test concentration ranges that were optimized to obtain more accurate LC25 and NOAEL. Ceritinib was re-tested at concentrations of 5, 10, 15, 20, and 25 μM and warfarin at concentrations of 10, 25, 50, 75, and 100 μM. Cyproheptadine hydrochloride was re-tested at concentrations of 5, 10, 25, 50, and 100 μM, and cyclobenzaprine hydrochloride at concentrations of 62.5, 125, 250, 500, and 1000 μM. In addition, because the categorized positive teratogenic compound aspirin showed cardiovascular toxicity at 2 dpf but this toxicity was completely recovered at 5dpf, it was rested at lower concentrations of 10, 25, 50, 75, and 100 μM. The concentration–mortality curve was calculated using OriginPro 8.0 software (Zheng et al., 2020) to obtain the best LC_25_ and NOAEL at 5 dpf and, correspondingly, a more realistic TI value. These compounds re-tested at refined concentrations were judged as teratogens if TI at 5 dpf was ≥ 3.

### Verification Tests of the Optimized Zebrafish Developmental Toxicity Assay

After the validation and optimization of the classical protocol, we selected three compounds to test their developmental toxicity and teratogenicity in zebrafish using our optimized protocol and standards developed from this study. Zebrafish were treated with triptolide, aconitine, or chlorogenic acid at concentrations of 0.1, 1, 10, 100, and 1000 μM, and LC_25_ and NOAEL at 2 dpf and 5 dpf were calculated based on their respective lethality curve. After getting an initial TI value, the testing concentrations were further refined and optimum TI values were obtained.

### Comparison to Human Data

The ICH Reference Compound List was selected based on data available in 2017 and derived from Detection of Toxicity to Reproduction for Human Pharmaceuticals. We further identified the relevant literature on the developmental toxicity of most selected compounds from searches of the PubMed (Medline) database. References were also identified from databases such as the Developmental and Reproductive Toxicology Database and the Hazardous Substances Data Bank. The results from the zebrafish developmental toxicity ftests were compared with human teratogenicity data in the ICH Harmonized Guideline S5(R3) (International Conference on Harmonization [ICH], 2017, 2020), and then the overall predictability of the zebrafish developmental toxicity and teratogenicity evaluation was obtained.

A compound was considered to be a developmental toxicant in conventional mammalian studies (rat, mouse, and rabbit, etc.) if it caused an increase in the occurrence of one of four manifestations: functional deficits, altered growth, structural abnormalities, or death (Schwetzm and Harris, 1993). In case conflicting conclusions were reported, we assumed the compound in question to be teratogenic. Literature shows that, for most compounds, data were available on teratogenicity on different strains of a species or laboratory animals’ development in multiple species. The overall concordance of this report was obtained by calculating the sensitivity (teratogens), specificity (non-teratogens), and overall predictability between human/mammal and zebrafish data (Selderslaghs et al., 2012).

### Assay Performance and Data Analysis

The study was conducted in accordance with the Basic & Clinical Pharmacology & Toxicology policy for experimental and clinical studies (Pernille et al., 2018). Successful experiments met all the following quality control milestones: (1) zebrafish natural death in untreated and vehicle-treated groups was ≤ 10%; (2) there was no statistical difference (*p* > 0.05) in assessed endpoints or signals between untreated and vehicle-treated groups; (3) intra- and inter-group coefficient of variation (CV) was ≤ 25%.

Mortality data were imported into Origin (OriginPro, version 8.0, 2007) and fitted to a sigmoidal equation with variable slope, thus creating concentration–response curves. These concentration–response curves were required to determine NOAEL and LC_25_ values. TI was calculated as the ratio of LC_25_/NOAEL for each time point. The correct classifications of positive and negative predictive values were imported into GraphPad (GraphPad Prism, version 5.0, 2003) for chi-square test, and *p* < 0.05 was considered significant.

The assay’s performance was evaluated by overall concordance. True positive (Y) and true negative (N) compounds were compounds that had zebrafish teratogenicity classification consistent with mammalian data. False-positive (FP) and false-negative (FN) were compounds that had zebrafish teratogenicity classification inconsistent with mammalian results. Uncategorized (U) meant compounds that induced visually observable malformation(s) on zebrafish, but TI values were <10. The analyses included determining the following endpoints: sensitivity for detecting teratogens = Y/(Y + FN + U) × 100%; specificity for detecting non-teratogens = N/(N + FP) × 100%; and overall concordance = (Y + N)/45 × 100%.

## Results

### Developmental Toxicity of the ICH Reference Compounds in Zebrafish as Validation of the Classical Protocol

Developmental toxicity of 45 ICH categorized positive (teratogenic) or negative (non-teratogenic) compounds was assessed at 2 dpf to 5 dpf zebrafish using parameters presented in Tables 3, 4, and Supplementary Tables 2–4, and LC_25_, NOAEL, and TI were calculated as described in Materials and Methods.

**TABLE 3:** Developmental toxicity results of positive controls (teratogen) compounds in zebrafish.

Compound	**2 dpf**			**5 dpf**			**Teratogenicity in zebrafish**		**Correct prediction**
	**LC_25_ (μM)**	**NOAEL(μM)**	**TI**	**LC_25_ (μM)**	**NOAEL (μM)**	**TI**	**Before optimization**	**After optimization**	
Diltiazem hydrochloride	>1000	>1000	1	>1000	100	>10	Y	Y	√
Topiramate	>1000	10	>100	>1000	100	>10	Y	Y	√
Phenytoin	>1000	>1000	1	>1000	10	>100	Y	Y	√
Carbamazepine	>1000	100	>10	>1000	100	>10	Y	Y	√
Aspirin	>100	10	>10	>10	>10	1	FN	Y*	√
Enalapril	>1000	>1000	1	>100	10	>10	Y	Y	√
Captopril	>100	>100	1	>100	10	>10	Y	Y	√
Methimazole	>1000	100	>10	>1000	100	>10	Y	Y	√
Dexamethasone	>1000	>1000	1	>1000	1	>1000	Y	Y	√
Cyclophosphamide	>1000	>1000	1	>1000	100	>10	Y	Y	√
Busulfan	>100	>100	1	>100	10	>10	Y	Y	√
Cisplatin	>100	>100	1	>100	10	>10	Y	Y	√
Acitretin	>100	<0.1	>1000	<0.1	<0.1	1	FN	Y*	√
Isotretinoin	>10	<0.1	>100	>0.1	<0.1	>1	FN	Y*	√
Theophylline anhydrous	>1000	100	>10	>1000	100	>10	Y	Y	√
Bosentan	>1000	>1000	1	>1000	10	>100	Y	Y	√
Artesunate	>100	1	>100	>1	0.1	>10	Y	Y	√
Clarithromycin	>1000	>1000	1	>1000	100	>10	Y	Y	√
Doxycycline hyclate	>100	>100	1	>100	10	>10	Y	Y	√
Fluconazole	>1000	>1000	1	>1000	100	>10	Y	Y	√
Afatinib dimaleate	>100	>100	1	>100	10	>10	Y	Y	√
Ceritinib	>10	>10	1	>10	>10	1	FN	Y(U)^#^	√
Dasatinib	>100	10	>10	>100	0.1	>1000	Y	Y	√
Pazopanib	>1000	1	>1000	>1000	1	>1000	Y	Y	√
Cytarabine	>1000	>1000	1	>1000	>1000	1	FN	FN	×
5-Fluorouracil	>1000	>1000	1	>1000	1	>1000	Y	Y	√
Hydroxyurea	>1000	>1000	1	>1000	>1000	1	FN	(U)	×
Methotrexate	>100	100	>1	>100	10	>10	Y	Y	√
Ribavirin	>1000	>1000	1	>1000	>1000	1	FN	FN	×
Teriflunomide	>1	1	>1	>1	0.1	>10	Y	Y	√
Warfarin	>10	>10	1	>10	>10	1	FN	Y(U)^#^	√

Y = yes, FN = false negative, and U = uncategorized. √: correct prediction; × : incorrect prediction. (U): A uncategorized compound judged only based on the TI value at Step 2 of the optimization. *: A false-negative compound was corrected to a teratogenic compound after adding an additional TI at 2 dpf along with the original TI at 5 dpf. #: After refining testing concentration range and optimizing TI parameters, a false-negative or uncategorized compound was finalized as a teratogenic compound.

**TABLE 4:** Developmental toxicity results of negative controls (non-teratogen) compounds in zebrafish.

**Compound**	**2 dpf**			**5 dpf**			**Teratogenicity in zebrafish**		**Correct prediction**
	**LC_25_ (μM)**	**NOAEL(μM)**	**TI**	**LC_25_ (μM)**	**NOAEL(μM)**	**TI**	**Before optimization**	**After optimization**	
Chlortalidone	>1000	>1000	1	>1000	>1000	1	N	N	√
Hydrochlorothiazide	>1000	>1000	1	>1000	>1000	1	N	N	√
Vildagliptin	>1000	>1000	1	>1000	>1000	1	N	N	√
Progesterone	>10	>10	1	>10	>10	1	N	N	√
Cetirizine hydrochloride	>100	>100	1	>100	>100	1	N	N	√
Cyproheptadine hydrochloride	>10	1	>10	10	1	10	FP	FP	×
Doxylamine succinate	>1000	>1000	1	>1000	>1000	1	N	N	√
Metoclopramide	>1000	>1000	1	>1000	>1000	1	N	N	√
Nizatidine	>1000	>1000	1	>1000	>1000	1	N	N	√
Clindamycin hydrochloride	>1000	>1000	1	>1000	>1000	1	N	N	√
Erythromycin	>1000	>1000	1	>1000	>1000	1	N	N	√
Amoxicillin	>1000	>1000	1	>1000	>1000	1	N	N	√
Sulfasalazine	>1000	>1000	1	>1000	>1000	1	N	N	√
Cyclobenzaprine hydrochloride	>1000	>1000	1	>100	10	>10	FP	FP	×

*N = no, FP = false positive. √: correct prediction; × : incorrect prediction.*

Based on the methods described in Step 1 in Materials and Methods, as shown in the results of “Before optimization” in Tables 3, 5, of 31 ICH-positive compounds, 23 compounds (74.2%) were teratogenic in zebrafish, and eight had false-negative results; of 14 ICH-negative compounds, 12 compounds (85.7%) were non-teratogenic in zebrafish and two had false positive results, and overall concordance was 77.8% (35/45). Table 4 contains a total of 14 negative controls, and the effects of 12 non-teratogenic compounds on zebrafish were highly consistent with the classification of ICH, except cyproheptadine hydrochloride and cyclobenzaprine hydrochloride, which were classified as negative controls by ICH but presented as false-positive results in zebrafish tests (Supplementary Figure 1). Cyproheptadine hydrochloride produced significant teratogenic phenotypes, including pericardial edema, bradycardia, absent blood flow, oversized jaw, small eyes, liver degeneration, yolk sac absorption delay, renal edema, and swim bladder loss, at a concentration of 10 μM with 25% zebrafish death and 100% death at 100 and 1000 μM. Cyclobenzaprine hydrochloride induced apparent liver degeneration and oversized jaw at 100 μM with 8.3% death and 100% death at 1000 μM.

**TABLE 5:** Comparison of prediction accuracy of zebrafish developmental toxicity assay before and after optimization.

**Results of judgment**		**Before optimization**	**After optimization**
Positive compounds	Test compounds	31	31
	Predictability consistent	23	28
	Predictability inconsistent	8	3
	*False negative compounds*	*8*	*2*
	*Uncategorized compounds*	*-*	*1*
	Sensitivity for detecting teratogens	74.2%	90.3%
Negative compounds	Test compounds	14	14
	Predictability consistent	12	12
	Predictability inconsistent	2	2
	*False positive compounds*	*2*	*2*
	Specificity for detecting non-teratogens	85.7%	85.7%
Overall concordance of predictability		77.8%	88.9%

Meanwhile, Table 3 contains a total of 31 positive controls, and the effects of 23 teratogenic compounds on zebrafish were highly consistent with the classification of ICH, except that aspirin, acitretin, isotretinoin, ceritinib, hydroxyurea, warfarin cytarabine, and ribavirin compounds had false-negative results when TI was below 10, as shown in the results of “Before optimization” in Table 3. Repeated experiments were performed on all 10 false-positive and false-negative compounds, and the results remained reproducible.

The morphological and functional abnormalities induced by teratogenic compounds at 2 dpf to 5 dpf in zebrafish are shown in Supplementary Tables 2–4. The most observed defects were pericardial edema, bradycardia, oversized jaw, yolk sac absorption delay, renal edema, and missing swim bladder. Aspirin had a typical dysplastic phenotype of brain hemorrhage; phenytoin showed a tachycardia; whereas the zebrafish treated with carbamazepine and cisplatin were developmentally much delayed and still in the chorion at the end of treatment (5 dpf).

### Optimizations of the Zebrafish Developmental Toxicity Assay

#### Optimization for Detection Time and Definition for Uncategorized Compounds

We found that the TI value of acitretin was >1000 at 2 dpf, which was significantly teratogenic, but with TI = 1 at 5 dpf, it was finally judged as a teratogen; this was in line with the new teratogenicity standard—a TI value from either 2 dpf or 5 dpf greater than or equal to 10 represented a teratogenic compound, as set by us from this study. TI values of aspirin, acitretin, and isotretinoin at 2 dpf were greater than 10, 1000, and 100, respectively, and thus, they were all re-categorized as teratogens, whereas cytarabine and ribavirin were still false-negative based on TI values both at 2 dpf or 5 dpf and morphology.

Ceritinib caused a toxic reaction manifested as missing swim bladder at 10 μM and 100% death at 100 and 1000 μM concentrations, but TI was below 10; warfarin caused 75% swim bladder loss and 100% yolk sac absorption delay at 10 μM and 100% death at 100 and 1000 μM, but TI was below 10; and hydroxyurea caused 100% swim bladder loss at 1000 μM, but TI was also less than 10 (Supplementary Figure 2). Thus, hydroxyurea, warfarin, and ceritinib were categorized as uncategorized compounds (Table 3), based on TI values for three uncategorized compounds smaller than 10 at both 2 dpf and 5 dpf, but they all induced morphological abnormalities.

#### Test Concentration Refinement and TI Value Optimization

Optimum concentrations and TI values were performed for one positive compound, two uncategorized compounds, and two false-positive compounds. As indicated in Supplementary Figure 3, the TI value of the positive compound aspirin was optimized to 3.9 in the refined concentration test. Not any toxic phenotype was found at 10 μM; one zebrafish had pericardial edema, yolk sac absorption delay, and swim bladder loss at 25 μM; five zebrafish with the delayed yolk sac absorption, swim bladder loss, cardiovascular toxicity, and Renal edema; and 12 dead when treated at 75 μM.

After refining the concentrations and calculating curves, the TI values of uncategorized compounds ceritinib and warfarin were shown at 3.5 and 23.9, respectively. Developmental toxicity phenotypes of ceritinib demonstrate three zebrafish with yolk sac absorption delay and swim bladder loss at 15 μM, and 12 dead when treated at 20 mM. Warfarin had cardiovascular toxicity at 10 μM; and four were dead when treated at 50 mM. TI value in the zebrafish treated with ceritinib at 5 dpf was smaller than 10 but greater than 3, and warfarin was greater than 10. If TI cut-off value was lowered from 10 to 3 based on these new results from the refined concentration experiments, ceritinib and warfarin were re-categorized from uncategorized compounds to teratogens.

Under the refined testing concentrations, the TI values of two false-positive compounds cyproheptadine hydrochloride and cyclobenzaprine hydrochloride TI were obtained at 13.8 and 11.7, respectively. Obviously, even in the optimized concentration tests, the TI values of cyproheptadine hydrochloride and cyclobenzaprine hydrochloride were still ≥ 10, and thus, they were true or false positive in the zebrafish developmental toxicity assay.

In summary, as shown in Tables 3, 5, after optimizations using the above methods, the predictability for five positive compounds became consistent from being inconsistent. The sensitivity of the zebra assay for detecting teratogens significantly increased from 74.2% (23/31) to 90.3% (28/31), and the overall predictability of all positive and negative compounds reached 88.9% (40/45), which is 77.8% (35/45) before optimizations.

### Application of the Optimized Zebrafish Developmental Toxicity Assay

To verify the optimized zebrafish developmental toxicity assay, three non-ICH compounds were tested in zebrafish using the new protocol and standards. As demonstrated in Table 6 and Supplementary Table 5, at 2 dpf, no compound-related toxic phenotypes and deaths were seen in the zebrafish treated with triptolide at 0.1 μM, but 12 zebrafish had pericardial edema at 1 μM, and 12 zebrafish died at 10 μM. At 5 dpf, 0.1 μM of triptolide treatment led to 12 zebrafish renal edema, pericardial edema, and cardiovascular toxicity, and three zebrafish died and nine zebrafish showed deformities at 1 μM. Therefore, the LC_25_ was 1 μM and the NOAEL was <0.1 μM.

**TABLE 6:** Application of the Optimized Zebrafish Developmental Toxicity Assay for triptolide, aconitine, and chlorogenic acid.

	**2 dpf**						**5 dpf**						**Teratogenicity**
	**Concentration (μM)**	**Number of deaths**	**Morphological abnormality**	**LC_25_ (μM)**	**NOAEL (μM)**	**TI**	**Concentration (μM)**	**Number of deaths**	**Morphological abnormality**	**LC_25_ (μM)**	**NOAEL (μM)**	**TI**	**in zebrafish**
Triptolide	1	0	12 zebrafish not hatched and pericardial edema	>1	>0.1	>10	0.1	0	12 zebrafish renal edema, pericardial edema, cardiovascular toxicity	1	<0.1	>10	Y
	10	12	-				1	3	9 zebrafish not hatched and malformation				
Aconitine	100	0	1 zebrafish not hatched, 2 zebrafish pericardial edema, cardiovascular toxicity	>1000	10	>100	10	0	12 zebrafish renal edema, yolk sac absorption delay	>100	1	>100	Y
	1000	0	11 zebrafish not hatched, 7 zebrafish pericardial edema, cardiovascular toxicity				100	2	10 zebrafish renal edema, pericardial edema				
Chlorogenic acid	10	0	2 zebrafish not hatched	>100	10	>10	10	0	-	>100	10	>10	Y
	100	2	7 zebrafish not hatched				100	2	6 zebrafish swim bladder missing, 5 zebrafish yolk sac absorption delay				

At 2 dpf zebrafish, aconitine induced in two zebrafish pericardial edema, cardiovascular toxicity, and no death at 100 μM, but no compound-related dysplasia at 10 μM; and in seven zebrafish, pericardial edema, cardiovascular toxicity, and no death at 1000 μM. The LC_25_ was >1000 μM and the NOAEL was 10 μM. At 5 dpf, 10 μM aconitine treatment resulted in 12 zebrafish having renal edema and delayed yolk sac absorption; at 100 μM, two zebrafish died, 10 zebrafish had renal edema and pericardial edema, and the LC_25_ was >100 μM and the NOAEL was 1 μM.

**FIGURE 1:**
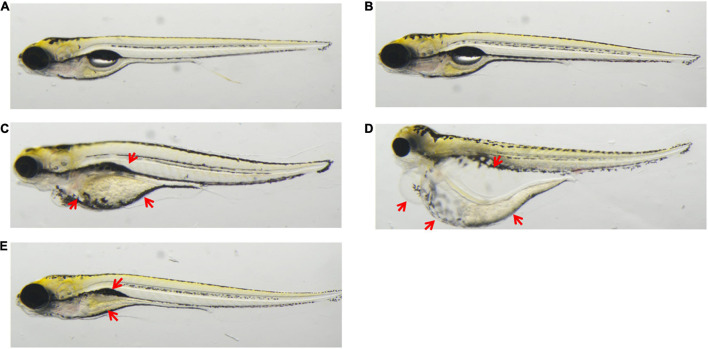
Application: triptolide, aconitine, chlorogenic acid, treatment to zebrafish at 5 dpf. **(A)** Untreated. **(B)** 0.5% DMSO. **(C)** 0.1 μM of triptolide treatment led to zebrafish renal edema, pericardial edema, and cardiovascular toxicity. **(D)** 100 μM of aconitine treatment led to zebrafish having renal edema and pericardial edema. **(E)** 100 μM of chlorogenic acid treatment led to zebrafish showing swim bladder loss and delayed yolk sac absorption.

At 2 dpf zebrafish, no compound-related toxicity was found from chlorogenic acid treatment and deaths were seen at 10 μM; at 100 μM, two zebrafish died, but no other dysplasia was observed, and the LC_25_ was >100 μM, and the NOAEL was 10 μM. At 5 dpf, no toxicity was detectable at 10 μM; at 100 μM, two zebrafish died, six zebrafish showed swim bladder loss, and five zebrafish had the delayed yolk sac absorption, and the LC_25_ was >100 μM and the NOAEL was 10 μM. The TI values of triptolide, aconitine, and chlorogenic acid were >10, >100, and >10, respectively, and all these three compounds were categorized as teratogenic agents in zebrafish. These results were further confirmed with the refined concentration tests as indicated in Figure 1 and Table 4.

## Discussion

Zebrafish have similar physiology, morphology, and functions with mammals and have been recognized as valuable *in vivo* models for toxicity and safety assessment of drug candidates and chemicals (Dooley and Zon, 2000; Mcgrath and Li, 2008; Macrae and Peterson, 2015). In recent years, zebrafish developmental toxicity assays and zebrafish embryotoxicity tests have been reported from several laboratories in the United States and Europe with inconsistent experimental methods, parameters, and sensitivity of prediction (Table 1). Most zebrafish developmental toxicity assays require the exposure of zebrafish embryos to up to 5 dpf for viability and morphology assessment. According to the European Union Directive (2010) on protection of laboratory animals, independent feeding is the stage at which zebrafish embryos are subject to regulations for animal experimentation (Gustafson et al., 2012; Strahle et al., 2012). Conventionally, post-hatched embryos >5 dpf are considered protected since the swim bladder is inflated, enabling free swimming and self-feeding (Belanger et al., 2010). Therefore, the design of this whole-organism assay is in compliance with definitions associated with the use of non-protected species (Schwetzm and Harris, 1993; Gustafson et al., 2012).

With reference to the internationally reported zebrafish developmental toxicity and teratogenicity evaluation methods, we intended to validate and optimize a quick and reliable alternative method for the IND regulatory use hopefully in China and in the world. Step 1 mainly referred to the method of AstraZeneca et al. reported in 2012 and 2014 (Gustafson et al., 2012; Ball et al., 2014). This method has a high throughput and saves new drug research and development time and cost; but in our validation study, this classical protocol had eight false negatives and the correct predictability was only 74.2%. We found that: (1) only using TI values at 5 dpf in the classical protocol could result in false-negative results. For example, isotretinoin as a relatively strong teratogenic drug induced a high percentage of zebrafish death at 5 dpf and, thus, affected TI value calculation (lower TI value). In addition, we and others found that the zebrafish cardiovascular system was more sensitive to toxic agents at 2 dpf (Zhu et al., 2014); in this study, for example, aspirin induced pericardial edema at 2 dpf, but this cardiovascular toxicity was recovered at 5 dpf; (2) testing concentration range at a 10 × scale in the classical protocol could quickly screen a large number of compounds in one test, but it could be easy to lose some classes of compounds with a narrow safety window, leading to false-negative results. For example, ceritinib induced zebrafish malformation, but its LC_25_ and NOAEL values were quite close; (3) that TI ≥ 10 as a teratogenicity cutoff as proposed in the classical protocol could mistakenly categorize some positive teratogenic compounds into negative ones and a smaller TI cut-off value could be more reasonable.

Based on our experience in the zebrafish toxicity and safety assays and the initial validation data, we added a new TI value at 2 dpf and compounds were assessed as teratogenic if either one or both TI values at 2 dpf and 5 dpf were greater than the cut-off value. This newly added TI at 2 dpf could help reduce false negatives.

There were only two categories of teratogenic and non-teratogenic compounds in the classical protocol, some compounds with TI values smaller than 10 at 2 dpf and 5 dpf but inducing toxic phenotypes could not be categorized as teratogenic or non-teratogenic. Therefore, we defined these neither teratogenic nor non-teratogenic compounds in the classical protocol assay as uncategorized compounds. In the further optimization study, we found that these uncategorized compounds could be divided into two sub-categories. For the first sub-category of uncategorized compounds, their LC_25_ and NOAEL both were greater than 1000 μM, i.e., no observable effects on zebrafish at 1000 μM; LC_25_ of these compounds was too high to be assessed for their developmental toxicity in zebrafish, and other types of animal tests could be needed. For the second sub-category of uncategorized compounds with LC_25_ or NOAEL smaller than 1000 μM, we performed new experiments using testing concentrations at smaller scales and obtained optimum TI values. After optimizing the TI cut-off value from ≥ 10 to ≥ 3, the second sub-category of uncategorized compounds was re-categorized into positive (teratogenic) compounds.

After these optimizations, the zebrafish developmental toxicity assay sensitivity was improved from 74.2% (23/31) to 90.3% (28/31), and the overall concordance was from 77.8% (35/45) to 88.9% (40/45). According to the ECVAM (European Center for the Validation of Alternative Methods) criteria for assessing the predictive value of a new assay (Genschow et al., 2002; Piersma et al., 2004), the assay was ranked excellent (>85%) for identifying developmental toxicity and teratogenic compounds.

In addition to obtaining the TI values, the morphological observations of developmental toxicity and teratogenicity played an important role in the judgment of drug toxicity, especially those drug-related morphological abnormalities. For the NOAEL biomarkers, we summarized the commonly and rarely observed morphological abnormalities of the zebrafish developmental toxicity and teratogenicity in Table 2. From our experience, if the zebrafish only showed a delayed yolk sac absorption and/or swim bladder loss after a compound treatment at 2 dpf and/or 5 dpf, these two malformations might not be used as NOVEL cutoff as we did in this study. We postulated that delayed yolk sac absorption and/or swim bladder loss were most likely related to developmental retard, but not teratogenicity.

After the validation and optimization of the classical protocol, we selected three non-ICH compounds (triptolide, aconitine, and chlorogenic acid) from our customer compound library isolated from Chinese herbs and tested them in the zebrafish developmental toxicity assay using the optimized protocol. Triptolide was toxic to human and male mouse reproductive systems (Ni et al., 2008) and aconitine was embryotoxic to rats *in vitro* (Xiao et al., 2007). To our best knowledge, the developmental toxicity of chlorogenic acid was unknown yet. Our data demonstrated that these three compounds were all teratogenic in zebrafish; the results of triptolide and aconitine in zebrafish were consistent with literature, whereas the results of chlorogenic acid should be confirmed in other tests in future investigations.

We must point out that the 45 ICH categorized as positive (teratogenic) and negative (non-teratogenic) compounds used in this study were selected from the ICH Reference Compound List for qualifying alternative assays in the S5(R3) draft guideline published in 2017 (International Conference on Harmonization [ICH], 2017). In this draft version, there were 16 negative compounds and 50 positive compounds listed. However, in the ICH final version promulgated in February 2020 (International Conference on Harmonization [ICH], 2020), only three negative compounds and 29 positive compounds were recommended, and the teratogenic effects of each of them on rats, rabbits, and humans were clarified. In 14 negative compounds tested in the current study, there were only two compounds, cetirizine and vildagliptin, included in the final version and they were also non-teratogenic in zebrafish. In 31 selected positive compounds, 18 compounds were in the 2020 version. Of these 18 positive compounds, 15 compounds were teratogenic and three compounds (cytarabine, ribavirin, and hydroxyurea) could not be assessed as teratogens in zebrafish. These results suggest that the zebrafish developmental toxicity assay may not be suitable for some types of compounds, or compound delivery through injection into zebrafish may be needed. Further investigations of compound structure–activity relationships and the assay optimization could clarify these and other issues. A challenging study is being planned to determine whether similar concordance could be reached with a larger set of pharmaceutical compounds and with mass spectrometry to quantify compound stability, absorption, metabolism and excretion.

As indicated in ICH S5(R3), the use of qualified alternative assays, such as non-mammalian *in vivo* assays, can reduce animal use while preserving the ability to detect relevant reproductive risks, and can be an appropriate approach for risk assessment under certain circumstances where they are interpreted in conjunction with routine *in vivo* reproductive testing. In addition, the use of qualified alternative assays can be a potential approach to defer *in vivo* testing as part of an integrated testing strategy. Our results in this study strongly support the zebrafish developmental toxicity assay as a predictable non-mammalian *in vivo* method for screening and assessing the teratogenicity of candidate compounds, and this zebrafish assay could be a promising alternative test system for regulatory acceptance. A multiple inter-laboratory validation study is being planned and will be reported in the future.

## Data Availability Statement

The original contributions presented in the study are included in the article/Supplementary Material, further inquiries can be directed to the corresponding author/s.

## Ethics Statement

The animal study was reviewed and approved by the zebrafish facility at Hunter Biotechnology, Inc. is accredited by the Association for Assessment and Accreditation of Laboratory Animal Care (AAALAC) International, the China National Accreditation Service for Conformity Assessment (CNAS), and the China Inspection Body and Laboratory Mandatory Approval (CMA).

## Author Contributions

Y-SS, L-JZ, YZ, and C-QL designed the research. M-ZD, Y-FH, and YP performed the research. Y-SS, M-ZD, Y-FH, YP, C-DZ, JL, and FX analyzed the data. M-ZD, Y-SS, C-XZ, L-JZ, and C-QL wrote the manuscript. All authors contributed to the article and approved the submitted version.

## Conflict of Interest

M-ZD, Y-FH, YP, YZ, and C-QL are employed by Hunter Biotechnology, Inc., which is mainly specialized in developing and marketing novel and innovative *in vivo* zebrafish-based assays for the following industries worldwide: Pharmaceutical, Biotechnology, Environmental, Natural Products, Industrial Chemical Products, Agrochemical, Food Additives, Skin Beauty Products, and Nutraceuticals. The remaining authors declare that the research was conducted in the absence of any commercial or financial relationships that could be construed as a potential conflict of interest.

## Publisher’s Note

All claims expressed in this article are solely those of the authors and do not necessarily represent those of their affiliated organizations, or those of the publisher, the editors and the reviewers. Any product that may be evaluated in this article, or claim that may be made by its manufacturer, is not guaranteed or endorsed by the publisher.
