# Supplementary material for: Validation, Optimization, and Application of the Zebrafish Developmental Toxicity Assay for Pharmaceuticals Under the ICH S5(R3) Guideline
Source: Front Cell Dev Biol. 2021 Sep 14;9:721130. doi: 10.3389/fcell.2021.721130 (PMC8476914; doi:10.3389/fcell.2021.721130)
Supplement: Supplementary file 1 [file Table_1.docx]

Supplementary Material

# Supplementary Figures and Tables

## Supplementary Figures


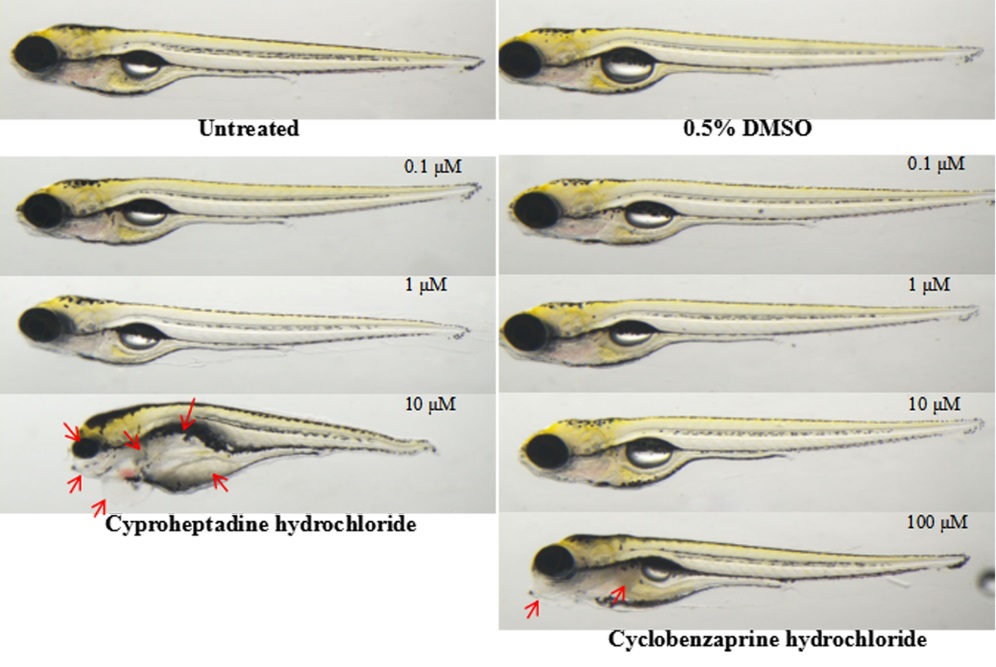


**Supplementary Figure 1. Developmental toxicity in zebrafish treated with 2 false positive compounds.** Cyproheptadine hydrochloride induced visually observable toxicity as arrows indicated, including pericardial edema, bradycardia, oversized jaw, small eyes, liver degeneration, yolk sac absorption delay, renal edema, swim bladder loss, at a concentration of 10 μM with 25% zebrafish death, and 100% death at 100 and 1000 μM. Cyclobenzaprine hydrochloride treatment led to apparent liver degeneration and oversized jaw at 100 μM with 8.3% death, and 100% death at 1000 μM.


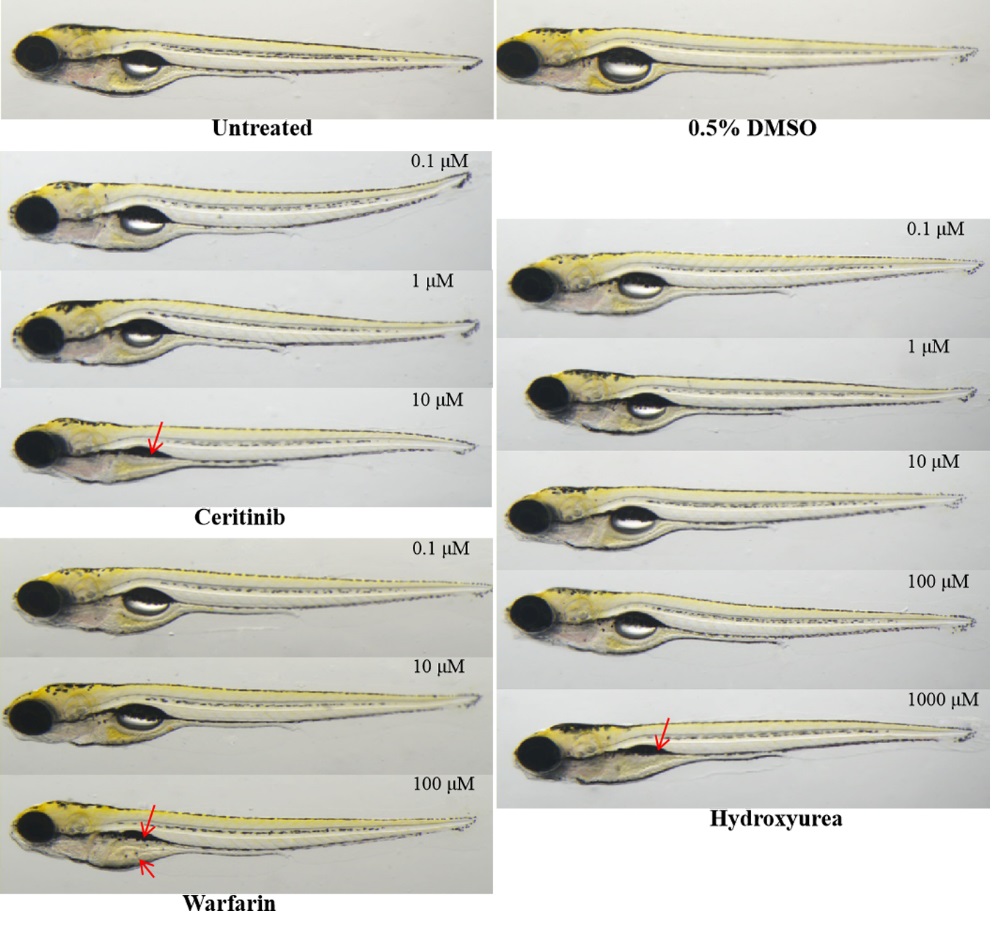


**Supplementary Figure 2.** **Treatment with 3 uncategorized compounds resulted in morphological abnormalities in zebrafish, as arrows indicated, but TI were <10.** Ceritinib caused a toxic reaction manifested as the missed swim bladder at 10 μM and 100% death at 100 and 1000 μM. Hydroxyurea caused swim bladder loss at 1000 μM. Warfarin caused swim bladder loss and yolk sac absorption delay at 10 μM, 100% death at 100 and 1000 μM.


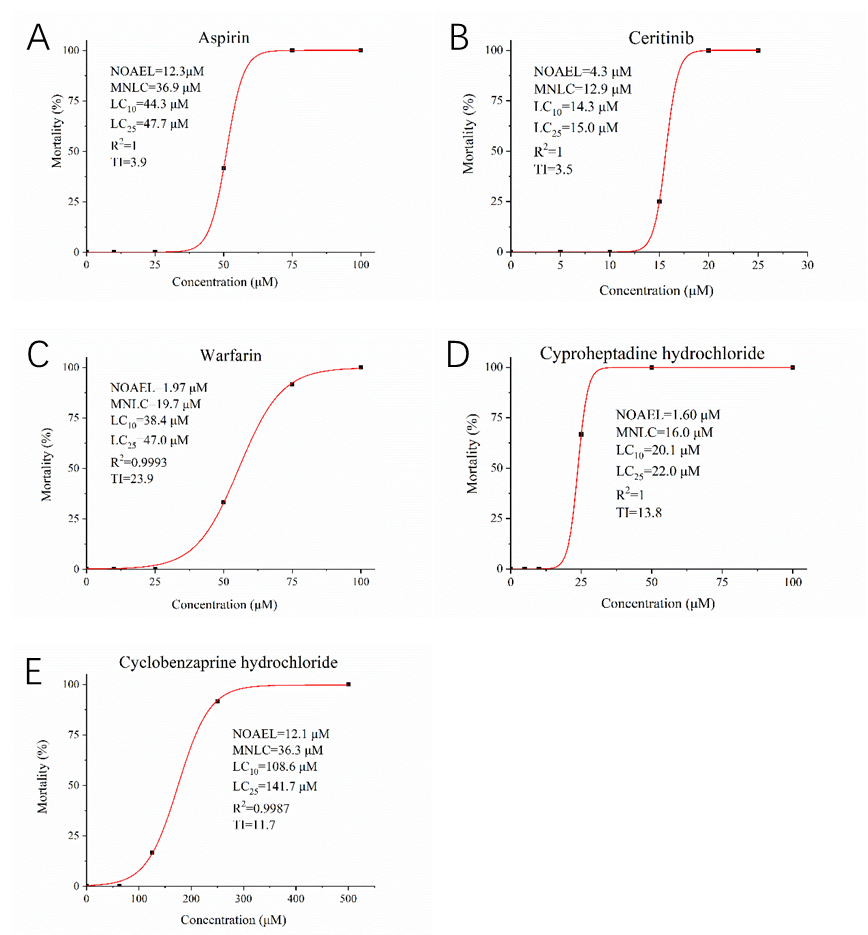


**Supplementary Figure 3.** Concentration refinement and concentration-mortality effect curves of 1 positive (A- Aspirin), 2 uncategorized (B- Ceritinib, C- Warfarin)，and 2 false positive compounds (D- Cyproheptadine hydrochloride, E- Cyclobenzaprine hydrochloride).

## 1.2 Supplementary Tables

**Supplementary Table 1. Information of selected 45 ICH compounds tested in zebrafish.**

| Compound | Purity | CAS-NO | Supplier | Category* |
| --- | --- | --- | --- | --- |
| Diltiazem hydrochloride | 98.99% | 33286-22-5 | Aladdin | Channel Modulator |
| Topiramate | 99.60% | 97240-79-4 | WD Pharma |  |
| Phenytoin | 99.40% | 57-41-0 | Aladdin |  |
| Carbamazepine | 99.49% | 298-46-4 | Aladdin |  |
| Chlortalidone | 98.9% | 77-36-1 | WD Pharma |  |
| Hydrochlorothiazide | 99.8% | 58-93-5 | WD Pharma |  |
| Aspirin | 99.9% | 50-78-2 | WD Pharma | Enzyme Modulator |
| Enalapril | 99.5% | 76095-16-4 | WD Pharma |  |
| Captopril | 99.6% | 62571-86-2 | WD Pharma |  |
| Methimazole | 99.21% | 616-47-7 | WD Pharma |  |
| Vildagliptin | 99.2% | 274901-16-5 | WD Pharma |  |
| Dexamethasone | 99.3% | 50-02-2 | WD Pharma | Hormone/Steroid |
| Progesterone | 99.30% | 57-83-0 | WD Pharma |  |
| Cyclophosphamide | 99.3% | 6055-19-2 | WD Pharma | DNA Modifiers |
| Busulfan | 99.77% | 55-98-1 | WD Pharma |  |
| Cisplatin | 99.25% | 15663-27-1 | WD Pharma |  |
| Acitretin | 99.9% | 55079-83-9 | WD Pharma | Transcription Modulator |
| Isotretinoin | 99.4% | 4759-48-2 | WD Pharma |  |
| Theophylline anhydrous | 99.5% | 58-55-9 | WD Pharma | Second Messenger Modulator |
| Bosentan | 99.6% | 157212-55-0 | WD Pharma | Receptor Modulator |
| Cetirizine hydrochloride | 99.5% | 83881-52-1 | WD Pharma |  |
| Cyproheptadine hydrochloride | 99.6% | 41354-29-4 | WD Pharma |  |
| Doxylamine succinate | 98.87% | 562-10-7 | Aladdin |  |
| Metoclopramide | 99.68% | 364-62-5 | WD Pharma |  |
| Nizatidine | 99.53% | 76963-41-2 | Aladdin |  |
| Artesunate | 98.3% | 182824-33-5 | WD Pharma | Others |
| Clarithromycin | 98.00% | 81103-11-9 | Aladdin |  |
| Doxycycline hyclate | 90.1% | / | WD Pharma |  |
| Fluconazole | 99.6% | 86386-73-4 | WD Pharma |  |
| Clindamycin hydrochloride | / | 21462-39-5 | WD Pharma |  |
| Erythromycin | 95.6% | 114-07-8 | WD Pharma |  |
| Amoxicillin | 100.2% | 26787-78-0 | WD Pharma |  |
| Sulfasalazine | 99.46% | 599-79-1 | WD Pharma |  |
| Cyclobenzaprine hydrochloride | 98.33% | 6202-23-9 | Aladdin |  |
| Afatinib dimaleate | 99.2% | 850140-72-6 | WD Pharma | Kinase Modulator |
| Ceritinib | 99.3% | 1030900-25-6 | WD Pharma |  |
| Dasatinib | 99.98% | 302962-49-8 | WD Pharma |  |
| Pazopanib | 99.95% | 444731-52-6 | WD Pharma |  |
| Cytarabine | 99.95% | 147-94-4 | WD Pharma | Nucleoside Modulator/  Central metabolite inhibitor |
| 5-Fluorouracil | 99.72% | 51-21-8 | WD Pharma |  |
| Hydroxyurea | 99.58% | 127-07-1 | WD Pharma |  |
| Methotrexate | 99.65% | 59-05-2 | WD Pharma |  |
| Ribavirin | / | 36791-04-5 | Aladdin |  |
| Teriflunomide | 99.00% | 163451-81-8 | Aladdin |  |
| Warfarin | 99.6% | 129-06-6 | WD Pharma |  |

* The categories were based on *in vivo* data from ICH.

**Supplementary Table 2. The morphological and functional abnormalities induced by compounds in zebrafish**

| Compound | Major defects | | | | | | | | |
| --- | --- | --- | --- | --- | --- | --- | --- | --- | --- |
|  | pericardial edema | bradycardia | absent or slow blood flow | hemorrhage | oversized jaw | liver degeneration | yolk sac absorption delay | renal edema | swim bladder missing |
| ICH positive compounds | | | | | | | | | |
| Diltiazem hydrochloride | + | + | + | - | - | + | - | - | - |
| Topiramate | + | + | - | - | - | - | + | - | + |
| Phenytoin | - | tachycardia | - | - | - | - | - | - | - |
| Carbamazepine | + | + | + | - | - | - | + | + | + |
| Aspirin | + | + | + | + | - | - | + | + | + |
| Enalapril | - | - | - | - | - | - | + | - | + |
| Captopril | - | - | - | - | - | - | + | - | + |
| Methimazole | - | - | - | - | + | + | + | + | - |
| Dexamethasone | + | + | + | - | + | - | + | + | + |
| Cyclophosphamide | - | - | - | - | - | - | + | - | + |
| Busulfan | - | - | - | - | - | - | + | - | + |
| Cisplatin | + | - | + | - | - | - | + | + | + |
| Acitretin | + | + | + | + | + | + | + | + | + |
| Isotretinoin | - | - | - | - | + | - | + | - | + |
| Theophylline anhydrous | - | - | + | - | + | + | + | + | - |
| Bosentan | - | - | - | - | - | + | - | - | - |
| Artesunate | - | + | + | - | + | - | + | + | + |
| Clarithromycin | + | - | - | + | - | - | + | - | + |
| Doxycycline hyclate | - | - | - | - | + | - | + | - | + |
| Fluconazole | - | - | - | - | - | + | - | - | - |
| Afatinib dimaleate | - | - | - | - | - | - | + | - | + |
| Dasatinib | + | + | + | + | + | + | + | + | - |
| Pazopanib | + | + | + | - | + | absent liver | + | + | + |
| 5-Fluorouracil | - | + | + | - | + | - | + | + | + |
| Methotrexate | + | + | - | - | - | - | - | + | - |
| Teriflunomide | - | - | - | - | - | - | + | - | + |
| Ceritinib | - | - | - | - | - | - | - | - | + |
| Hydroxyurea | - | - | - | - | - | - | - | - | + |
| Warfarin | - | - | - | - | - | - | + | - | + |
| ICH negatives-compound | | | | | | | | | |
| Cyproheptadine hydrochloride | + | - | - | - | + | - | + | + | + |
| Cyclobenzaprine hydrochloride | - | + | + | - | + | + | - | + | - |

+ morphological/functional characteristic was affected; - morphological/functional characteristic was not affected.
