# Supplementary material for: Validation, Optimization, and Application of the Zebrafish Developmental Toxicity Assay for Pharmaceuticals Under the ICH S5(R3) Guideline
Source: Front Cell Dev Biol. 2021 Sep 14;9:721130. doi: 10.3389/fcell.2021.721130 (PMC8476914; doi:10.3389/fcell.2021.721130)
Supplement: Supplementary file 3 [file Table_3.DOCX]

**Supplementary Table 6. The physical and chemical properties of the ICH compounds**

|  | Category | Positive/negative  controls from ICH | Compound | CAS-NO | Melting  point/range | Appearance | pH (at 1000 μM) | Information Source |
| --- | --- | --- | --- | --- | --- | --- | --- | --- |
| 1 | Channel Modulator | Positive Controls  (Teratogen) | Diltiazem hydrochloride | 33286-22-5 | 207, 5 - 212 ℃  212212 °C | Powder; White | 5.05 | <https://www.sigmaaldrich.cn/CN/en/sds/sigma/d2521> |
|  |  |  | Topiramate | 97240-79-4 | - | Solid | 5.40 | <https://www.sigmaaldrich.cn/CN/en/sds/sigma/t0575> |
|  |  |  | Phenytoin | 57-41-0 | 293 - 295 °C - lit. | Solid | 5.26 | <https://www.sigmaaldrich.cn/CN/en/sds/sigma/d4007> |
|  |  |  | Carbamazepine | 298-46-4 | 191 - 192 °C | Powder; White | 5.33 | <https://www.sigmaaldrich.cn/CN/en/sds/sial/94496> |
|  |  | Negative Controls  (Non-teratogen) | Chlortalidone | 77-36-1 | 265 - 267 °C | Solid | 5.46 | <https://file.medchemexpress.cn/batch_PDF/HY-15833/Chlorthalidone-SDS-MedChemExpress.pdf> |
|  |  |  | Hydrochlorothiazide | 58-93-5 | 265 - 267 °C | Solid; White | 5.34 | <https://www.sigmaaldrich.cn/CN/en/sds/sial/h2910> |
| 2 | Enzyme Modulator | Positive Controls  (Teratogen) | Aspirin | 50-78-2 | 138 - 140 °C | Crystalline; White | 3.53 | <https://www.sigmaaldrich.cn/CN/en/sds/sial/phr1003> |
|  |  |  | Enalapril | 76095-16-4 | 143 - 144,5 °C | Solid | 3.05 | <https://www.sigmaaldrich.cn/CN/en/sds/sial/e0110000> |
|  |  |  | Captopril | 62571-86-2 | 104 - 108 °C - lit. | Powder; White | 3.20 | <https://www.sigmaaldrich.cn/CN/en/sds/sigma/c4042> |
|  |  |  | Methimazole | 616-47-7 | -6 °C | Liquid; Clear | 7.68 | <https://www.sigmaaldrich.cn/CN/en/sds/sial/336092> |
|  |  | Negative Controls  (Non-teratogen) | Vildagliptin | 274901-16-5 | 148 - 150 °C | Solid | 7.27 | <https://www.sigmaaldrich.cn/CN/en/sds/sigma/sml2302> |
| 3 | Hormone/  Steroid | Positive Controls  (Teratogen) | Dexamethasone | 50-02-2 | - | Powder | 5.27 | <https://www.sigmaaldrich.cn/CN/en/sds/sigma/d4902> |
|  |  | Negative Controls  (Non-teratogen) | Progesterone | 57-83-0 | - | Solid | 5.35 | <https://www.sigmaaldrich.cn/CN/en/sds/aldrich/903744> |
| 4 | DNA Modifiers | Positive Controls  (Teratogen) | Cyclophosphamide | 6055-19-2 | 49 - 51 °C | solid | 5.05 | <https://www.sigmaaldrich.cn/CN/en/sds/mm/239785> |
|  |  |  | Busulfan | 55-98-1 | 114 - 117 °C - lit. | Crystalline; Beige | 4.58 | <https://www.sigmaaldrich.cn/CN/en/sds/sial/b2635> |
|  |  |  | Cisplatin | 15663-27-1 | 340 °C | Solid | 5.84 | <https://file.medchemexpress.cn/batch_PDF/HY-17394/Cisplatin-SDS-MedChemExpress.pdf> |
| 5 | Transcription Modulator | Positive Controls  (Teratogen) | Acitretin | 55079-83-9 | 228 - 230 °C | Solid | 5.27 | <https://www.sigmaaldrich.cn/CN/en/sds/sigma/44707> |
|  |  |  | Isotretinoin | 4759-48-2 | 174 - 175 °C | Solid | 5.00 | <https://www.sigmaaldrich.cn/CN/en/sds/sial/phr1188> |
| 6 | Second Messenger | Positive Controls  (Teratogen) | Theophylline anhydrous | 58-55-9 | - | Solid | 5.28 | <https://file.medchemexpress.cn/batch_PDF/HY-B0809/Theophylline-SDS-MedChemExpress.pdf> |
| 7 | Receptor Modulator | Positive Controls  (Teratogen) | Bosentan | 157212-55-0 | 114 - 118 °C - Dec. | Solid | 5.25 | <https://www.sigmaaldrich.cn/CN/en/sds/usp/1076115> |
|  |  | Negative Controls  (Non-teratogen) | Cetirizine hydrochloride | 83881-52-1 | 225 °C | Solid | 2.76 | <https://www.sigmaaldrich.cn/CN/en/sds/usp/1102929> |
|  |  |  | Cyproheptadine hydrochloride | 41354-29-4 | - | Solid | 6.38 | <https://www.sigmaaldrich.cn/CN/en/sds/usp/1161000> |
|  |  |  | Doxylamine succinate | 562-10-7 | 103 - 108 °C | Solid | 4.79 | <https://www.sigmaaldrich.cn/CN/en/sds/sial/y0001562> |
|  |  |  | Metoclopramide | 364-62-5 | 147 °C | Solid | 8.90 | <https://www.sigmaaldrich.cn/CN/en/sds/sial/m1824990> |
|  |  |  | Nizatidine | 76963-41-2 | 130 - 132 °C | Solid | 7.10 | <https://www.sigmaaldrich.cn/CN/zh/sds/sigma/sml2960> |
| 8 | Other | Positive Controls  (Teratogen) | Artesunate | 182824-33-5 | - | Solid | 5.42 | - |
|  |  |  | Clarithromycin | 81103-11-9 | - | Solid | 7.58 | <https://www.sigmaaldrich.cn/CN/en/sds/sigma/sbr00011> |
|  |  |  | Doxycycline hyclate | / | - | - | 3.15 | - |
|  |  |  | Fluconazole | 86386-73-4 | 138 - 140 °C | Solid | 5.28 | <https://www.sigmaaldrich.cn/CN/en/sds/sial/y0000558> |
|  |  | Negative Controls  (Non-teratogen) | Clindamycin hydrochloride | 21462-39-5 | - | Crystalline; White | 5.10 | <https://www.sigmaaldrich.cn/CN/en/sds/sigma/c5269> |
|  |  |  | Erythromycin | 114-07-8 | 133 °C | Solid | 8.21 | <https://www.sigmaaldrich.cn/CN/en/sds/sial/e1305000> |
|  |  |  | Amoxicillin | 61336-70-7 | 140°C | Solid | 4.46 | https://www.chemicalbook.com/ProductChemicalPropertiesCB3690305.htm |
|  |  |  | Sulfasalazine | 599-79-1 | 260 - 265 °C - dec. | Crystalline | 3.88 | <https://www.sigmaaldrich.cn/CN/en/sds/sial/s0883> |
|  |  |  | Cyclobenzaprine hydrochloride | 6202-23-9 | 216 - 218 °C | Solid | 5.17 | <https://www.sigmaaldrich.cn/CN/en/sds/sigma/c4542> |
| 9 | Kinase Modulator | Positive Controls  (Teratogen) | Afatinib dimaleate | 850140-72-6 | - | Solid | 3.86 | <https://www.sigmaaldrich.cn/CN/en/sds/sigma/sml3109> |
|  |  |  | Ceritinib | 1030900-25-6 | - | Solid | 8.04 | - |
|  |  |  | Dasatinib | 302962-49-8 | - | Solid | 6.73 | <https://www.sigmaaldrich.cn/CN/en/sds/sigma/sml2589> |
|  |  |  | Pazopanib | 444731-52-6 | - | Solid | 5.54 | <https://www.sigmaaldrich.cn/CN/en/sds/aldrich/cds023580> |
| 10 | Nucleoside Modulator/  Central metabolite inhibitor | Positive Controls  (Teratogen) | Cytarabine | 147-94-4 | - | Crystalline; White | 5.86 | <https://www.sigmaaldrich.cn/CN/en/sds/sial/phr1787> |
|  |  |  | 5-Fluorouracil | 51-21-8 | 282 - 286 °C - dec. | Powder | 5.28 | <https://www.sigmaaldrich.cn/CN/en/sds/sigma/f6627> |
|  |  |  | Hydroxyurea | 127-07-1 | 135 °C | Solid | 3.64 | <https://www.sigmaaldrich.cn/CN/en/sds/sial/y0000119> |
|  |  |  | Methotrexate | 59-05-2 | 195 °C | Solid | 5.45 | <https://www.sigmaaldrich.cn/CN/en/sds/sigma/m7824> |
|  |  |  | Ribavirin | 36791-04-5 | - | Solid | 5.00 | <https://www.sigmaaldrich.cn/CN/en/sds/sigma/r9644> |
|  |  |  | Teriflunomide | 163451-81-8 | 229 -232 °C | Solid | - | https://www.chemicalbook.com/chemicalproductproperty_cn_cb02486749.htm |
|  |  |  | Warfarin | 129-06-6 | 161 °C | - | 4.41 | <https://www.sigmaaldrich.cn/CN/en/sds/aldrich/a4571> |
